# The effect of load on spatial statistical learning

**DOI:** 10.1038/s41598-023-38404-2

**Published:** 2023-07-20

**Authors:** Nadav Amsalem, Tomer Sahar, Tal Makovski

**Affiliations:** 1 Department of Education and Psychology, The Open University, The Dorothy de Rothschild Campus, 1 University Road, P. O. Box 808, 43107 Ra’anana, Israel; 2 School of Psychological Sciences, University of Haifa, Haifa, Israel

**Keywords:** Human behaviour, Spatial memory

## Abstract

Statistical learning (SL), the extraction of regularities embedded in the environment, is often viewed as a fundamental and effortless process. However, whether spatial SL requires resources, or it can operate in parallel to other demands, is still not clear. To examine this issue, we tested spatial SL using the standard lab experiment under concurrent demands: high- and low-cognitive load (Experiment 1) and, spatial memory load (Experiment 2) during the familiarization phase. We found that any type of high-load demands during the familiarization abolished learning. Experiment 3 compared SL under spatial low-load and no-load. We found robust learning in the no-load condition that was dramatically reduced in the low-load condition. Finally, we compared a no-load condition with a very low-load, infrequent dot-probe condition that posed minimal demands while still requiring attention to the display (Experiment 4). The results showed, once again, that any concurrent task during the familiarization phase largely impaired spatial SL. Taken together, we conclude that spatial SL requires resources, a finding that challenges the view that the extraction of spatial regularities is automatic and implicit and suggests that this fundamental learning process is not as effortless as was typically assumed. We further discuss the practical and methodological implications of these findings.

## Introduction

Our visual world is full of dynamic and ever-changing incoming stimuli. Given the vast amount of visual input, one of the challenges is to cope with the stimuli load and prepare for the expected stimuli. Fortunately, objects in the environment often appear not in a random fashion but rather in certain repeated contexts. Extracting and utilizing these regularities in the environment is an excellent tool to reduce the amount of information needed to be processed and allow a more efficient allocation of resources. Indeed, such extraction of regularities is widely accepted as a fundamental cognitive process^1,2^ that occurs implicitly, without direct intention or awareness, effortlessly, and without perturbing other concurrent processes^3^. That is, while explicit visual memory relies on limited capacity and available resources and is constrained by divided attention^4–9^, implicit learning of those visual regularities should, by definition, only be minimally affected, if at all, by other concurrent cognitive demands.

One such form of implicit visual learning is visual *Statistical Learning* (SL)^2,10–12^. A visual SL task commonly consists of two parts; First, participants view a stream of complex stimuli, usually composed of arbitrary shapes. Unbeknown to them, some of the stimuli occur in a certain temporal regularity, and those regularities repeat within blocks of trials. After the learning (or familiarization) phase, a test phase is administered. Participants view two streams of shapes—old (stimuli that appeared in the same temporal regularity as in the familiarization phase) and new (stimuli that appeared in a different temporal order), and the task is to decide which one of the two is more familiar. Importantly, both the old and new streams consist of pairs or triplets composed of the complex stimuli seen in the learning phase and thus performance above chance level indicates that the observer was not merely familiar with certain stimuli but incidentally learned the regularities: the specific temporal associations among the stimuli. To conclude that the learning occurred implicitly, some studies also directly test participants’ explicit knowledge about the repeated regularities^12–17^.

**Figure 1:**
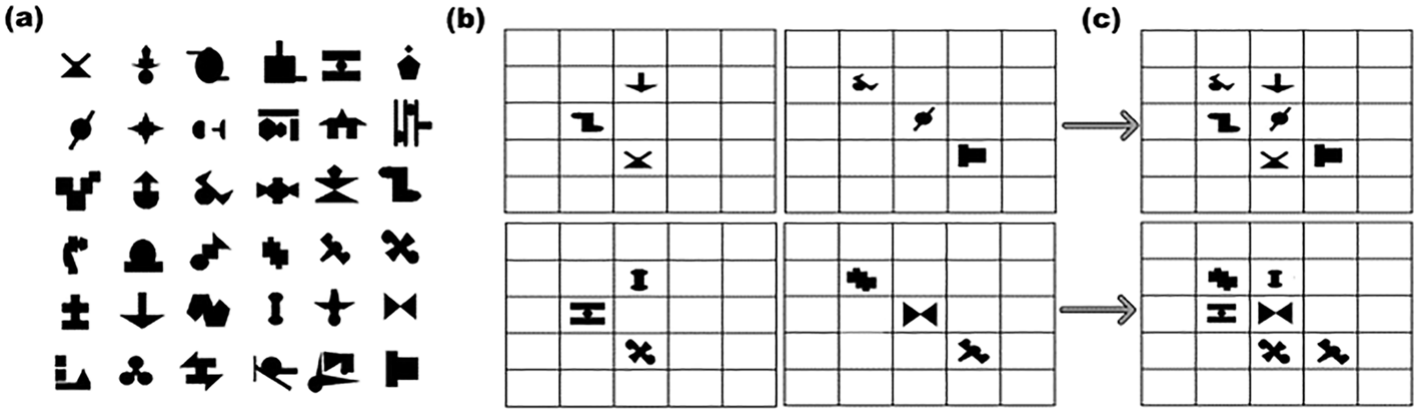
Illustration of Stimuli: (**a**) The spatial SL shapes used in all experiments (**b**) Illustration of base triplets (**c**) Illustration of the spatial SL display used in all experiments.

Participants can learn not only temporal regularities^18^ but spatial ones as well^14,16,19,20^. In a seminal study^19^, participants saw displays with a complex spatial arrangement of six shapes. Critically, these arrangements were created by laying out pairs or triplets of specific shapes, in a specific spatial configuration (see below, Fig. 1b). Participants viewed each complex display only once, but in fact, each of the six base pairs was repeated in 72 trials. Again, the test phase consisted of a base (old stimuli) and foil (new stimuli) discrimination task, and participants were asked to indicate which of the two was more familiar. The results from three experiments showed implicit, unaware, and non-intentional extraction of spatial statistics.

These findings illustrate the remarkable ability to extract non-trivial spatial and temporal relationships among visual (and auditory^11^) arbitrary stimuli, mostly occurring without direct intention to acquire them. Describing this notion, authors have often used the term *automaticity*^15,21,22^ wrapping several aspects of SL together: (a) *incidental*—learning occurs without direct intention, (b) *unawareness*—learning occurs without explicit awareness of the regularities or without awareness that such information was acquired, (c) *fundamental process*—the extracted information does not depend on specific stimuli, and the information is available for other ongoing processes, (d) *inattentional*—learning does not depend on the allocation of attention, and, (e) *effortless*—learning does not depend on available resources and occurs with little or no cost on other processes^3^. Yet, as described below, evidence for the effortless aspect of SL is mixed.

In the absence of additional demands, temporal SL can operate robustly^21^ but is somewhat limited under additional demands^23^. Recent findings point out that task demands during the learning phase affect temporal SL^24,25^ but critically, it is not known how concurrent demands affect learning in both the temporal and spatial SL forms.

Other types of implicit learning tasks suggest that implicit learning in the spatial domain is quite robust in the face of additional demands. For instance, a series of experiments^26^ examined how different types of visual working memory tasks (e.g., color arrays, dot location arrays) affected a simple visual search task (i.e., T among L task). Importantly, in their search task, the target location was either cued explicitly (by spatial or verbal cues) or implicitly (by a high probability that the target will appear at a certain quadrant of the display). They found that any concurrent memory task that created a load impaired only explicit cues, but not implicit cues extracted through incidental learning, see also^27^.

Another study demonstrated that implicit learning is not easily affected by concurrent demands^28^. This study examined whether contextual cueing, a visual search task where the target location is implicitly or incidentally learned through the repeated configuration of the target and its surrounding distractors^29–31^ is affected by memory load from concurrent working memory tasks. They found that contextual cueing was robust to concurrent demands and suggested that implicit context learning does not rely on visual or spatial working memory resources (but see^32,33^). Interestingly, however, when a spatial memory task was administered during the testing phase (rather than the learning phase) the contextual cueing effect was impaired, suggesting that concurrent demands impaired the expression of learning but not to the learning itself^34,35^.

To the best of our knowledge, no other study has directly examined the effects of load (by concurrent tasks) on visual SL. This question has two important implications; from a practical standpoint, most visual SL tasks employ some sort of a cover task during the SL learning phase. That is, participants are not told to memorize the SL displays directly, but rather to perform an adjacent task like repetition detection, to ensure that participants are actively engaged in the display. Inadvertently, these demands, as weak as they might appear, could distort, or impair learning. More importantly, from a theoretical standpoint, if load affects SL, then it is not as effortless as previously assumed. In the current study, we focused on the spatial, rather than the temporal, version of the task since it is easier to manipulate load on a trial-by-trial basis, and as abovementioned it is not clear how load affects this form of SL. Our goal was therefore to examine how different types of load (i.e., concurrent additional demands) during the learning phase affect the outcome of incidental spatial SL.

## Experiment 1

The goal of Experiment 1 was to test whether incidental learning of spatial regularities is robust to memory load during the familiarization phase. In each trial, participants performed a letters-digits old-new memory task (simple characters change-detection task). During the retention interval of the memory task, we showed participants a 6-shapes spatial SL display. Half of the trials in the task were low-load and half high-load, and different base triplets were associated with each condition. Thus, we were able to examine if the subsequent recognition of base triplets is affected by the amount of load (high vs. low conditions) during familiarization.

## Method

### Participants

Participants in all experiments were The Open University of Israel undergraduate students who took part in the experiments for course credit (age range: 18–40). All had normal or corrected vision, normal color vision, and without any neurological or attention deficits. The sample size was based on a previous study^14^ with 20 participants showing a large effect of learning (Cohen’s d > 0.8, see Exp. 3 below). Taking into consideration that we introduced a load manipulation, we set the minimal sample size of 35 participants. Power analysis (*pwr* package for r, 2018, version 1.2-2, http://cran.r-project.org/web/packages/pwr/) confirmed that this sample size provides 95% power to detect an effect size of at least 0.5 (Cohen’s d) with two-sided paired samples t-test. Forty-seven participants completed Experiment 1 (Mean age = 26.5, 31 females, 16 males).

### Materials

The task was programmed and administered using MATLAB software (MATLAB, The MathWorks Inc., Natick, MA, 2010) and Psychtoolbox on a Standard PC and 23.5″ LCD Eizo Foris monitor (1920 × 1080, 120 Hz refresh rate). Analyses were performed using JASP (version 0.15, JASP team, 2021). Bayesian analyses were performed with the Standard prior (Caucy ~ 0.707). The stimuli were 24 black-and-white shapes obtained from Turk-Browne’s (2005) study^22^. Each shape (1.14° × 1.14°) was presented within a cell of a black visible grid (5 × 5, 11.4° × 11.4°), on a white background. For the memory task, we used the digits 0–9 and the A–J letters (font: Segoe UI, 32 points 0.57° × 1.05°).

### Spatial SL display

The 24 stimuli were randomly sampled for each participant: 12 unique shapes for the high memory load condition and 12 unique shapes for the low memory load condition (Fig. 1a). The 12 shapes were then randomly divided into four base triplets. Thus, each load condition had four unique base triplets. Two of the four were two left arrow-shaped triplets and the other two were left diagonal triplets (Fig. 1b). The spatial SL display was a combination of one arrow triplet and one diagonal triplet presented together and therefore each load condition had four unique SL displays. Each spatial SL display was composed of six stimuli arranged in a combination of arrow and diagonal triplets positioned at the nine central grid cells. The other grid cells remained empty (Fig. 1c).

### Procedure

**Figure 2:**
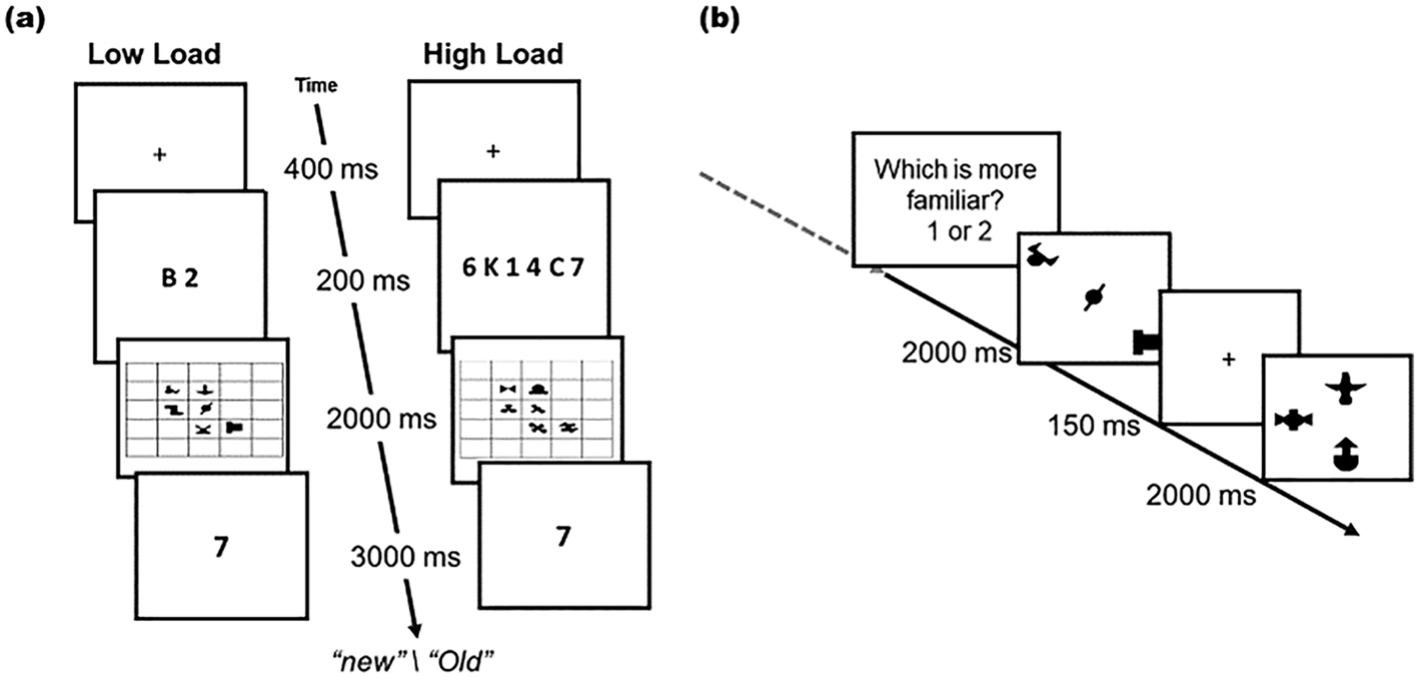
Schematic Illustration of Experiment 1: (**a**) Illustration of Experiment 1’s trial sequences during the learning phase in the low (left sequence)—and high-load (right sequence) conditions. (**b**) Schematic illustration of the spatial SL familiarity task. Note: the triplets appeared on a 5 by 5 grid.

The procedure followed standard spatial SL tasks as there was a learning phase followed by a test phase (Fig. 2). Each trial sequence in the learning phase started with a fixation display (a black cross, 0.57°) presented for 400 ms. Then, the memory items display appeared for 200 ms. In the low-load condition of Experiment 1, one letter and one digit (each randomly chosen) appeared together at the center of the screen. In the high-load condition, a random combination of 3 letters and 3 digits appeared. The memory array was followed by a spatial SL display presented for 2 s. After viewing the spatial SL display, participants performed the memory test, which consisted of one “old” character (i.e., appeared), or one “new” character (i.e., did not appear). The test items were presented at the center of the screen (Fig. 2a). In each load condition, half of the trials were old, and half were new, randomly distributed across the blocks. Participants were instructed to respond whether the test item (i.e., one letter or digit) was old or new by pressing the corresponding keys (‘h’ or, ‘j’) on the keyboard within three seconds. A feedback display (11.4° × 11.4°, “correct”, “incorrect”, or “too slow”) appeared for 300 ms, followed by a blank interval of 600 ms. There were 112 trials in each load condition, divided into four blocks of 56 trials. The block sequence was ABAB (e.g., high-load, low-load, high-load, low-load) with their order counterbalanced across participants. For each load condition, there were four spatial SL displays, each repeated 28 times (thus, for each of the load conditions, a unique triplet repeated 56 times). Before the experimental trials, participants performed eight practice trials, four in each load condition, composed of random stimuli configuration (Fig. 1a). The order of SL displays, in both conditions, was randomly and evenly assigned across trials within each block.

Participants were instructed to perform the character memory task (i.e., to respond on each trial whether the test stimulus is “old” or “new”) and were not told anything about the SL display (which appeared as fillers during the retention interval in each trial). After completing the study phase, participants took a short break and received the instructions for the test phase from the experimenter. Each trial in the test phase (Fig. 2b) consisted of two triplets, presented one after the other on a center grid (5 × 5). Each triplet was presented for two seconds with 150 ms interval between them. One triplet was a triplet that appeared in the learning phase (i.e., old triplet), and another triplet consisted of three random shapes from that load condition (i.e., new triplet). Randomly, in half of these trials, the old triplet appeared first, and in the other half, it appeared second. Participants were asked to respond by pressing the ‘1’ or ‘2’ keys to which of the two triplets was more familiar. Each old triplet was tested four times; twice against each of the two ‘new’ foil triplets. The presentation order was evenly and randomly balanced for each old triplet. The foil triplets were always presented as the opposite shape to the target triplet; i.e., if the target triplet was a ‘diagonal’ (Fig. 1b right), then the foil was an ‘arrow-shaped’ triplet (Fig. 1b left), and vice-versa. Thus, the recognition test consisted of 16 trials (four old triplets, each tested four times) for each of the high- and low-load conditions in a random order, for a total of 32 trials.

### Ethical approval

This study was approved by the ethics committee of The Open University of Israel (3193), all participants consented prior to experiments. All experiments were performed in accordance to the guidelines and regulations of the Declaration of Helsinki.

### Conference presentation

Part of this work was submitted by NA to fulfill his requirements for a master’s thesis in Psychology (MA). This paper was presented at the conference for Object Perception, Attention, and Memory (OPAM, Nov. 2022, Boston, USA). A version of this manuscript available as a pre-print at https://doi.org/10.31234/osf.io/4wksj.

## Results and discussion

Seven participants whose accuracy in the memory task of the learning phase was two standard deviations from the mean in one of the two load conditions were excluded from the analyses. This left us with 40 participants’ data. Including the excluded participants did not change any of the conclusions (see Fig. 5 below, for a summary of the results).

### Character memory task

Performance in the memory task during the learning phase in the low-load condition (*M* = 93%, *SD* = 6.7) was, as expected, better that the high-load condition (*M* = 70%, *SD* = 10.1), *t*(39) = 17.7, *p* < 0.001, *d* = 2.8. A similar pattern was observed for mean correct RT, with faster performance in the low-load condition (*M* = 923 ms, *SD* = 238 ms) compared to the high-load condition (*M* = 1039 ms, *SD* = 244 ms), *t*(39) = 3.3, *p* = 0.002, *d* = 0.522.

### Spatial SL task

Learning was observed in the low-load condition. Performance was significantly larger than the chance level (a one-sided test that the mean is larger than 50%, *M* = 57%), *t*(39) = 3.2, *p* < 0.001, *d* = 0.506. In contrast, the high-load condition was not better than chance (*M* = 53%), *t*(39) = 0.935, *p* = 0.178, *BF*_01_ = 2.32, and was significantly lower than the low-load condition,* t*(39) = 2.22, *p* = 0.032, *d* = 0.351. Thus, Experiment 1 provided clear evidence that high memory load impaired the learning of spatial regularities.

## Experiment 2

**Figure 3:**
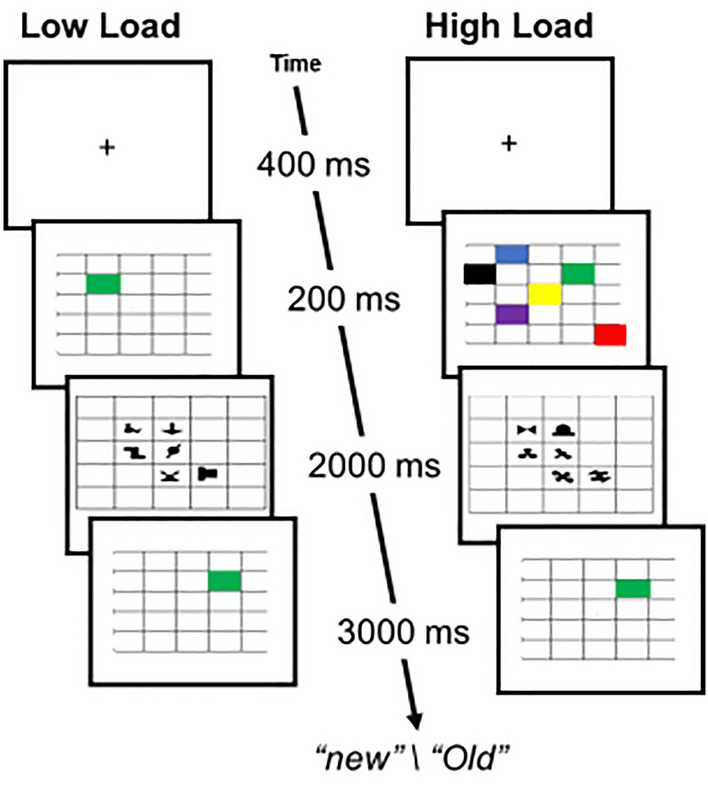
Schematic Illustration of Experiment 2. Illustration of Experiment 2’s trial sequences during the learning phase in the low (left sequence) and, in the high-load conditions (right sequence).

While Experiment 1 showed that visual memory load impaired spatial SL, it is not yet clear whether all types of memory load would behave the same. For instance, as mentioned above, the expression of the contextual cueing effect was impaired only under spatial memory load, and not by non-spatial load^34^. This compels to test the effect of load using a spatial memory task. This should allow us to generalize our conclusions and might even reveal a greater impairment stemming from the spatial nature of the task. To this end, the design of Experiment 2 was identical to Experiment 1’s, except that instead of the character memory task, low- and high-spatial memory load were introduced during the learning phase (Fig. 3).

## Method

The materials, procedure, and design were the same as in Experiment 1, except for the following changes. The memory items were colored squares (Fig. 2b, 0.91° × 0.91°) filled with black (RGB = 0,0,0), green (RGB = 0,255,0), blue (RGB = 0,0,255), yellow (RGB = 255,255,0), magenta (RGB = 255,0,255), or red (RGB = 255,0,0). In the low-load trials, one squared color appeared at a randomly chosen grid cell. In the high-load condition, six different colors simultaneously appeared in six different cells. Participants were instructed to respond whether the test stimulus, one old color item, was presented at the same location as before (“old”) or a different location (“new”). Old and new test stimuli were equally and randomly presented. Four participants whose performance exceeded 2 SDs in one of the load conditions were removed from the analysis leaving 37 participants (Mean age = 27.1, 30 females, 7 males) in the final sample.

## Results

### Spatial memory task

As in Experiment 1, performance in the memory task during the learning phase in the low-load condition (*M* = 94%, *SD* = 3.5) was more accurate than the high-load condition (*M* = 70%, *SD* = 10.7), *t*(36) = 15.2, *p* < 0.001, *d* = 2.54. Performance was also faster in the low-load condition (*M* = 900 ms, *SD* = 158 ms) than in the high-load condition (*M* = 1127 ms, *SD* = 249 ms).

### Spatial SL task

Akin to Experiment 1, only the low-load condition showed learning and accuracy was significantly larger than the 50% chance performance (*M* = 56%), *t*(36) = 2.57, *p* = 0.014, *d* = 0.424. The high-load condition was within the chance performance (*M* = 53%), *t*(36) = 1.574, *p* = 0.124, *BF*_01_ = 1.8, yet the high-load and the low-load were not significantly different, *t*(36) = 0.814, *p* = 0.421, *BF*_01_ = 4.1.

## Discussion

Like Experiment 1, we observed learning under the low-load condition, but not under the high-load condition. We can therefore conclude that learning is constrained under any type of high-load (i.e., memory or spatial). However, the support for the lack of significant learning in the high-load condition was anecdotal and the current results still need to be clarified. Specifically, in contrast to Experiment 1, the low- and high-spatial load conditions of Experiment 2 were not statistically different while the low-load was statistically larger than 50 percent chance performance (and the high-load was not). We believe that the lack of significant difference is the product of the rather small learning effect found even under the low load condition (56%). That is, when learning is quite poor, to begin with, it would be difficult to find the considerable difference between the two load conditions. In the next experiment, we addressed this issue by comparing a low-load condition to a no-load condition, which allowed us to estimate the magnitude of learning without any task.

## Experiment 3

**Figure 4:**
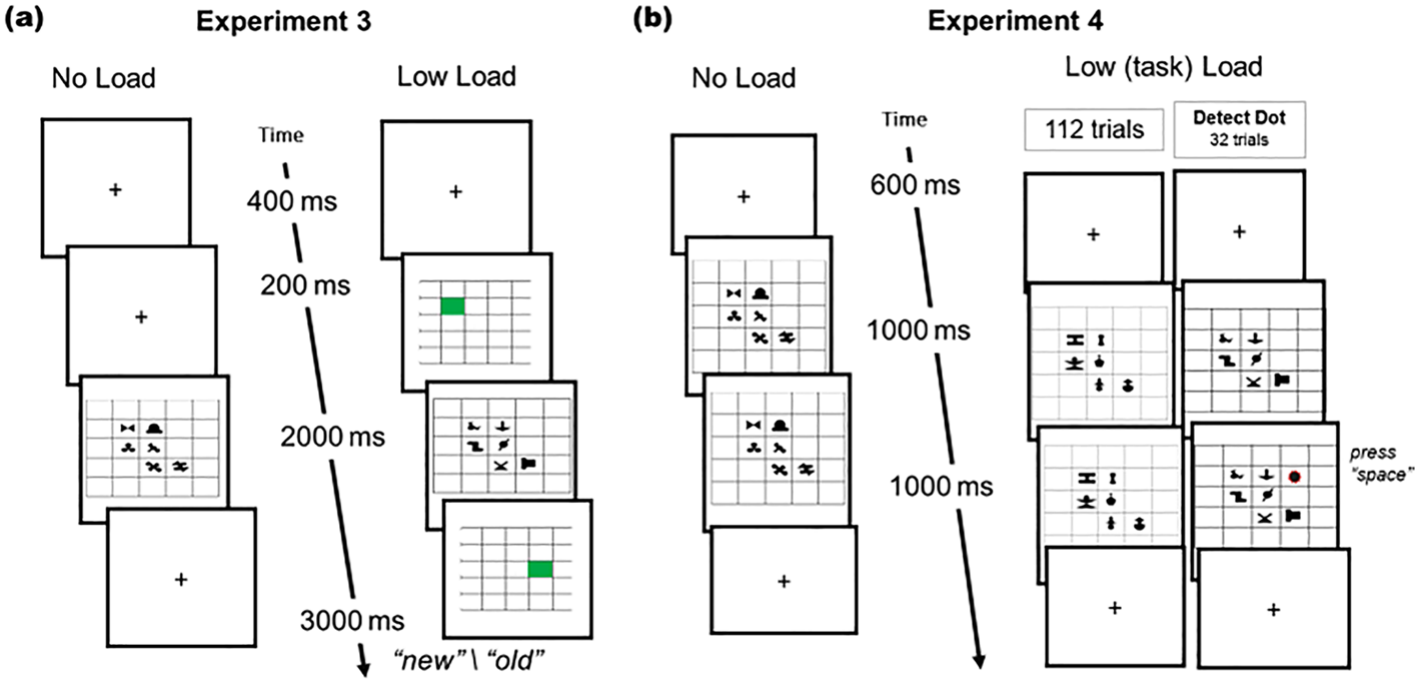
Schematic Illustration of Experiments 3 and 4 (**a**) Schematic illustration of Experiment 3 design: low-load trials (right) and no-load trials (left) (**b**) Schematic illustration of Experiment 4 design: task load trials with infrequent dot trials interleaved (“Detect dot”, right), and the no-load trials (left). Note: The dot color was black circled here in red for visualization.

The results of the first two experiments were consistent in showing no learning under the high-load conditions. However, the difference in learning between the low- and high-load conditions was relatively small and significant only in the first experiment. We suspected this could be because most of the load effect was introduced by the secondary task itself, which brought the learning down, leaving little room for additional load effect. Indeed, in their classical study, Fiser and Aslin^14^ reported a much greater learning effect (Exp. 1^14^: Cohen’s d ~ 1.2) when using a similar design, but without any additional secondary task^19^. Thus, in the next experiment, we compared spatial SL performance under two conditions: a no-load condition, in which participants viewed the repeating SL displays without performing any concurrent task, and a low-load condition with an easy spatial memory task during the learning phase (the same as in the low-load condition of Experiment 2, Fig. 4a). Directly comparing the low-load with the no-load condition allows us to quantify undisturbed learning, and how additional, even rather low load demands, affect learning.

## Method

The materials, procedure, and design were the same as in Experiment 2, except for the following changes. The task consisted of a low-spatial load condition, the same as in the low-spatial load condition of Experiment 2, and a no-load condition (i.e., passive-viewing) in which the participants were only required to passively view the spatial SL displays. Participants in the no-load condition were told just to observe the screen and displays and were not informed that there will be a test on these shapes. In the low-load condition, participants were instructed to perform the spatial memory task (as in Experiment 2) and were not told anything about the filler SL displays. Stimuli, trial design, number of trials, and the display sequence and timing were the same as the low-load task.

To minimize carry-over effects, the block’s sequence was now AABB (i.e., low-load, no-load) counterbalanced across participants. Forty-three new participants (Mean age = 30.4, 34 females, 10 males) completed Experiment 3.

## Results

### Spatial memory task

In the memory task during the learning phase, the mean correct responses was 94% (SD = 4.4, mean RT = 902 ms, SD = 221 ms). Accuracy was not significantly different from the low-load condition of Experiment 2 (*M* = 94%, independent samples test, *t*(78) = 0.019, *p* = 0.985, *BF*_*01*_ = 4.2).

### Spatial SL task

Both conditions were significantly larger than the chance level, suggesting that spatial SL was able to occur in parallel to the low-spatial load (*M* = 56%), *t*(42) = 4.12, *p* < 0.001, *d* = 0.629. Learning was found also in the no-load condition, (*M* = 70%), *t*(42) = 7.59, *p* < 0.001, *d* = 1.1), and most importantly it was far more robust than the low-load condition, *t*(42) = 4.89, *p* < 0.001, *d* = 0.746.

## Discussion

Experiment 3’s results replicated within the same participants, both the large magnitude of learning reported in the classical spatial SL effect^14^, and the smaller, still better-than-chance performance found in the low spatial load condition of Experiment 2. Taken together, performing even a low-load concurrent task considerably impaired the robustness of statistical learning of spatial regularities.

Still, one might argue that the no-load condition might have prompted participants to engage more with the SL displays, and therefore enhanced learning in this condition might result from the increased attention to the displays. We addressed this issue in the next experiment.

## Experiment 4

Experiments 1–3 showed that a concurrent task, even one which creates a low degree of load, impairs the learning of spatial regularities. Further increasing either cognitive or spatial load during that phase was detrimental to the learning. In contrast, the learning under the no-load condition, without any concurrent task, was robust. This entails both theoretical and practical implications as studies often use conditions in which participants are passively viewing the displays to measure SL. Yet, it is not clear what subjects are actually doing during the no-load condition. One possibility is that “doing nothing” encourages them to actively search for regularities. Thus, the goal of our final experiment was to address this issue by providing a cover task to minimize this ‘active search’ tendency while keeping the load at a minimum (if any at all).

To this end, instead of a low spatial load, we tested a condition in which participants were asked to respond as quickly as they could upon the detection of an un-frequent dot-probe on filler displays (see above, Fig. 4b). We reasoned that such a task should hardly impose any additional load while keeping participants engaged with a task. In fact, the ‘alertness’ for the detection of the dot by sustaining visual attention to the display might assist the learning. On the opposite, impaired learning in that condition relative to no-load would suggest that the presence of an additional task *itself*, even one that adds a minimal concurrent load impairs SL.

## Method

The materials, procedure, and design were similar to those of Experiment 3, except for the following changes. Because we needed more displays for the dot-probe detection task, we added 12 new arbitrary spatial SL shapes (created with common paint software) to the existing pool of shapes (stimuli available at https://osf.io/qbdwr/). The 36 shapes were randomly divided into three unique sets of 4 triplets. These sets were randomly created across participants. Importantly, each set was matched to a condition during the learning phase. The task consisted of a passive-viewing 112 trials like Experiment 3 no-load condition. In addition, there was a dot-probe condition (i.e., task load), where in 32 random trials across the 144 trials, a black dot (0.8° × 0.8°, RGB = 0,0,0) appeared on a randomly chosen empty grid-cell, one second into the spatial SL display. The display remained on-screen for an additional second and participants were asked to respond as fast as they could by pressing the spacebar when they saw the black dot. Participants were not told anything about the SL displays. Similarly, in the no-load condition, participants were not told anything about the SL displays, just to observe the screen until they get further instructions. Importantly, the spatial SL displays during the dot-probed trials were of four unique triplets which were not tested in the spatial SL test phase. The remaining 112 trials in that condition were of four unique triplets that did appear in the spatial SL test phase. The block’s sequence was AABB (i.e., task-load, no-load) counterbalanced across participants. Forty new participants (Mean age = 27.4, 23 females, 17 males) completed Experiment 4.

## Results and discussion

### Dot-probe task

Average dot detection hit rate was 98.4% (*SD* = 2.4). The mean detection RT for the dot probe trials was 680 ms (*SD* = 175 ms).

### Spatial SL task

As in Experiment 3, robust learning was found in the no-load condition (*M* = 75%), *t*(39) = 8.67, *p* < 0.001, *d* = 1.37. There was also robust learning in the dot-probe task condition, (*M* = 68%), *t*(39) = 6.5, *p* < 0.001, *d* = 1.02. The two conditions were statistically different *t*(39) = 3.11, *p* = 0.003, *d* = 0.492. Interestingly, recognition of SL triplets in the low-task load of Experiment 4 (*M* = 68%) was significantly more accurate than the low-spatial load of Experiment 3 (*M* = 57%, independent samples Welch t-test, *t*(65.2) = 3.39, *p* < 0.001, *d* = 0.751). This finding suggests that task load, which could harness resources onto the primary task display, might facilitate the learning of spatial regularities (compared to other cover tasks that might share demands with the learning). Nevertheless, other methodological parameters differ between the tasks (e.g., memory load and response on each trial, spatial load vs. dual-task load) and hence it is yet to be determined how exactly secondary task parameters affect learning. Importantly, there was still an advantage for learning spatial regularities when this learning occurred under a passive viewing condition without any additional demands.

## General discussion

**Figure 5:**
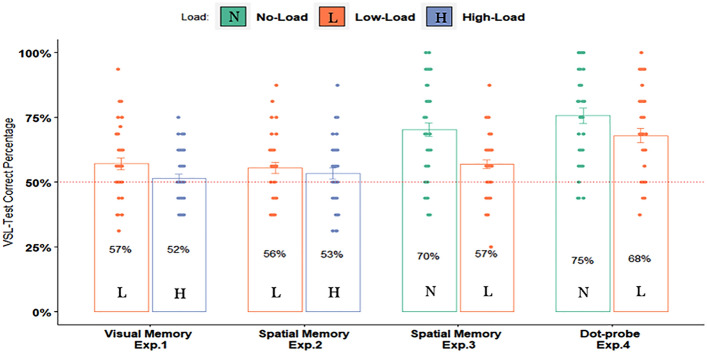
Summary of the results from Experiments 1–4. Percentage of correct responses in the spatial SL task as a function of the load condition and type of task. Error bars represent ± 1 standard error of the mean. The horizontal dotted line represents the chance level performance of 50% correct responses. Mean accuracy (Percent correct) is indicated in each bar. The colored dots represent individual scores. Note: L = Low-load condition, H = High-load condition, N = No-load condition.

In the present study, we examined how different load types during exposure to spatial SL displays affect the incidental learning of spatial regularities of arbitrary shapes. Figure 5 depicts a summary of the results.

As can be seen, we found that nearly any amount or type of concurrent load during the viewing of the spatial SL displays impaired the later recognition of the spatial relations among the shapes. We introduced a low memory load by a concurrent memory task of digits and letters (Experiment 1) and a low spatial load from the location of colored squares (Experiment 2). Both resulted in poor but above-chance levels of learning. Importantly, in both Experiments 1 and 2, any type of concurrent high-load during the viewing of the displays abolished the learning. We further replicated the relatively powerful spatial SL effect under a no-load, passive viewing condition (Experiment 3) and showed that it was dramatically reduced even under a low spatial load condition. Experiment 4 similarly replicated the large learning effect under a no-load condition showing again a benefit, this time over a simple, infrequent dot-probe cover task that required minimal demands. That is, even a simple task with minimal load (if any) that encouraged participants to pay attention to the display, impaired spatial SL. Notably, however, this cover task induced a much smaller impairment to SL when compared to the spatial low-load tasks confirming a gradual effect of load on learning. Together, these results are consistent with the general notion that task demands during exposure influence SL^23,24^ and further demonstrate that incidental learning of spatial relations is not an effortless process. This means that any use of resources directly shared or not disrupts SL.

While the temporal form of SL is more commonly used than spatial SL, the role of load in any of these forms was hardly directly tested. Temporal tasks often used a repetition detection (n-back task) during the learning phase^21,22^ or another detection task during the presentation of the stream^15,17,20^. Regardless of the amount of load it creates, we showed that the presence of a *task itself* during the learning, even without any specific working memory load, impaired spatial SL. Since it was shown that SL results in a flexible representation that can transfer between the spatial and temporal domains^20^ (i.e., learned temporal relations were expressed as spatial, and vice versa), it is reasonable to speculate with a degree of confidence that *temporal SL* should be affected by load much like the spatial form. Future research could directly examine the impact of load in the temporal domain (e.g., with an n-back task^36^). Importantly, the current results are clear that this type of SL is less resistant to parallel demands than previously suggested^15,22,37^.

In contrast to other types of implicit learning tasks, such as contextual cueing, which were found to be robust to load or dual-task situations^26,28,38,39^ our results demonstrated that load easily disrupted spatial SL. This discrepancy raises the possibility that spatial SL might not be “as implicit”^41^ as other visual tasks that are robust to concurrent demands^15^. Indeed, the interfering effects of load on a spatial SL task are aligned with the idea that some implicit learning tasks actually require some degree of explicit processes^40–43^.

Yet, before concluding that spatial SL is an explicit rather than implicit process we should first consider several differences between visual learning tasks that are affected by load (e.g., spatial SL) and those which are not (e.g., probability learning^26,27^, contextual cueing^28,30,34^). For instance, this discrepancy might stem from a fundamental difference in what is learned: target position vs. association between shapes. The latter, which is mostly irrelevant when searching for an item, might be more demanding and more susceptible to the effect of load. Another possibility is that the visual search task used in both probabilistic cueing and contextual cueing has an inherent “task load” to begin with, and therefore the effects of any additional demands might be more difficult to detect.

Our findings suggest that spatial SL is easily disturbed by other ongoing processes regardless of their nature. Whether SL in general is robust when it comes to operating in parallel to other demands^43^ is a critical and unresolved question. Moreover, as discussed above, the automaticity of SL can be viewed from different aspects^3,22^. Here, we focused on one aspect of automaticity, namely effortfulness. The other aspects of automaticity, such as the incidental nature of the task, and that observers often fail to report that they noticed a repetition or that they learned anything, are relatively undisputed. Thus, while we conclude that spatial SL might not be a purely implicit, effortless process^39,43^, one needs to be cautious in generalizing and comparing our results to other types of implicit learning tasks.

Despite several differences found between various types of SL tasks, the common findings of visual SL are often taken together even with findings from audition^44^ and language acquisition^45^ to support the claim that SL is a general-domain process^1^. It was further claimed that this general-domain SL is constrained by working memory limitations^1,46–48^. While the current study did not aim to directly test this notion, the findings are indeed consistent with it as we showed that SL is constrained by non-selective working memory demands (e.g., character memory and spatial color memory). Yet, the question of whether similar mechanisms are underlying SL of various modalities and domains is to be determined.

From a broader theoretical perspective, we argue the fact that people incidentally learn arbitrary, meaningless spatial configurations is quite remarkable even if this learning ability is easily disrupted by load. Furthermore, that participants were able to recognize non-trivial spatial relations among arbitrary shapes above chance performance suggests that contextual information, like spatial configurations, might be hard-wired into these memory representations^14,49–51^.

The current results also bear practical and methodological implications. From a practical perspective, there are several examples where one can think of applying spatial SL in real-world settings, such as learning the configuration of your apps on the smartphone, or remembering the sitting arrangement of students in your class. All these and others might benefit from trying to learn them actively and intentionally without any parallel task. Accordingly, future studies should examine SL and how it is affected by load in more ecological environments (e.g., using VR).

In terms of methodology, researchers should reconsider using passive viewing conditions for testing learning as it might overestimate both the magnitude and the implicit nature of learning^12,22,52^. On the one hand, there are good reasons to test passive viewing condition as it is likely to be more ecological since in everyday life we rarely engage in detection tasks^53^. On the other hand, it is not clear whether learning is automatic in the sense that it is imposed on the observer even when instructed to ignore the stimuli^21,54^. Instead, learning might be robust under this condition just because participants explicitly search for regularities out of experimental boredom (this condition was described as *“the most boring thing I have ever done”* by an anonymous participant), experimental curiosity, or just because they have nothing else to do.

Note that even if the no-load condition, in its passive viewing form, compelled participants to engage with the displays, then the enhanced learning under this condition is in a sense the result of more effort and resources invested in learning. This points to the positive consequences of directly engaging with the display^12,54,55^ rather than engaging with some other task. In any case, it is advisable to incorporate other, non-demanding cover tasks when testing implicit learning mechanisms. Future studies should search for the specific conditions that emulate no-load trials while controlling for participants’ engagement.

In conclusion, our study provides clear evidence that spatial SL requires resources, that learning is impaired as the task load during familiarization increases, and that a presence of a task, even minimally demanding, reduces learning. These findings critically challenge that SL occurs automatically and call for more caution in designing implicit learning cover tasks as those could largely impact learning.

## Data Availability

None of the experiments were formally preregistered. The data of the current study are available in the Open Science Framework (OSF) repository, https://osf.io/qbdwr/.
